# The application value of continuous nursing intervention on quality of life in patients with stroke: A protocol for systematic review and meta-analysis

**DOI:** 10.1097/MD.0000000000025963

**Published:** 2021-06-04

**Authors:** Cai-Yan Han, Xia Yu

**Affiliations:** a Department of Neurology; b Department of Pediatrics, Hanchuan People's Hospital, Hanchuan, Hubei Province, China.

**Keywords:** continuous nursing intervention, meta-analysis, quality of life, stroke

## Abstract

**Background:**

Individual characteristics, physical function disability, emotional, as well as cognitive symptoms, along with the general health discernment might be associated or impact the quality of life of patients suffering from stroke directly or indirectly. Appropriate continuous nursing intervention is required to enhance the quality of life of patients with stroke. Therefore, the present study will be conducted to systematically investigate the application value of continuous nursing intervention for improving the quality of life of patients experiencing stroke.

**Methods:**

We will conduct a comprehensive search of electronic databases such as MEDLINE, Cochrane Library, CINAHL, EMBASE, Scopus, Chinese National Knowledge Infrastructure, and WanFang databases to identify relevant publications. We will only include studies published in English or Chinese languages. Accordingly, randomized controlled trials evaluating the application value of continuous nursing intervention for improving the quality of life of patients suffering from stroke will be included. We will use 2 independent authors to conduct study selection, extract data, and evaluate the quality of the included studies. In case of any discrepancies, they will be addressed by consensus. Also, we will use RevMan 5.3 software to carry out the statistical analysis.

**Results:**

The current study will summarize high-quality evidence to systematically explore application value of continuous nursing intervention for improving the quality of life in patients with stroke.

**Conclusion:**

The present study will summarize the direct and indirect pieces of evidence to ascertain whether continuous nursing intervention can improve the quality of life in patients with stroke.

**Ethics and dissemination:**

Ethical approval will not be required.

**Registration number:**

April 25, 2021.osf.io/xnrzt/ (https://osf.io/xnrzt/).

## 1 Introduction

Stroke is regarded as one of the common conditions with higher incidence rates, estimated to occur at 76 to 119 for every 100,000 population every year; and result in higher mortality and disability rates among affected populations.^[1]^ Overall, the increasing prevalence and incidence rates of stroke are witnessed among aging populations. An estimate by some scholars suppose that the occurrence of stroke will be estimated to increase to approximately 3 times the current rate by 2030.^[2]^ Accordingly, stoke is estimated to cause many symptoms and can severely impair people's quality of life, causing “mental, physical, functional, and psychological disorders, and high cost of medical care.”^[3,4]^

Patients with stroke mainly induce cognitive and motor disturbances such as balance, muscle power, muscle tone, gait, proprioception, and coordination; these disturbances must be included into the rehabilitation process.^[5,6]^ Additionally, the recovery assessment of stroke should focus on these aspects. In regards to quality of life, in the context of a person's health condition, it is considered to be a health-related quality of life. Because the aspect of health entails multi-dimensional features, health-related quality of life also covers multi-dimensional domains that are linked to the social, physical, emotional, and psychological functions.^[7]^

Improving patients’ physical and mental health, along with their quality of life, it is needful to ascertain variables that can relate to stroke and manage factors that are already eminent. In conventional nursing, there are systematic educations as well as discharge guidance of disease-related knowledge in the period or process of hospitalization. This terminates the health guidance of patients following discharge. Still, with constant nursing, as an extension of high-quality medial services to the family, it is possible to comprehend the compliance behavior and treatment effect, or psychological state of a patient following their discharge from a healthcare institution. Generally, provision of medical and psychological guidance to the patients is critical to improving their quality of life.^[8,9]^ Therefore, the present study will be conducted to systematically investigate the application value of continuous nursing intervention for improving the quality of life among patients suffering from stroke.

## 2 Objectives

The present study aims to explore the application value of continuous nursing intervention for improving the quality of life of patients with stroke.

## 3 Methods

### 3.1 Study registration

We have registered the protocol on the Open Science Framework (OSF, http://osf.io/. The study will follow the Preferred Reporting Items for Systematic Review and Meta-Analysis Protocols.^[10]^

### 3.2 Eligibility criteria for included studies

#### 3.2.1 Types of participants

We will only include patients with confirmed diagnosis of stroke, without restrictions of age, gender, race, and country.

#### 3.2.2 Types of participants

We will administer continuous nursing intervention for patients in the experimental group and conventional nursing intervention or no nursing intervention for patients in the comparisons group.

#### 3.2.3 Types of outcomes

The major outcomes are Beck Depression Inventory, Fear of Stroke Recurrence Scale, degree of physical function disability, and Stroke-specific quality of life scale.

#### 3.2.4 Types of studies

We will use random assignment of participants to continuous nursing intervention groups and conventional nursing intervention group or no nursing intervention group.

### 3.3 Search methods for identification of studies

#### 3.3.1 Electronic searches

We will carry out a comprehensive search of electronic databases including MEDLINE, Cochrane Library, CINAHL, EMBASE, Scopus, Chinese National Knowledge Infrastructure, and WanFang databases to establish relevant publications from their inception to April 2021. We will include only studies published in English or Chinese languages. The following search terms will be combined using Boolean logic (AND, OR, or NOT) to identify relevant studies: “RCT,” “randomized controlled trial,” “randomized controlled trial,” “stroke,∗” “continuous nursing,” “quality of life.”

#### 3.3.2 Search other sources

In addition, we will search other sources to identify relevant publications related to continuous nursing intervention.

### 3.4 Data collection and analysis

#### 3.4.1 Study selection

**Figure 1:**
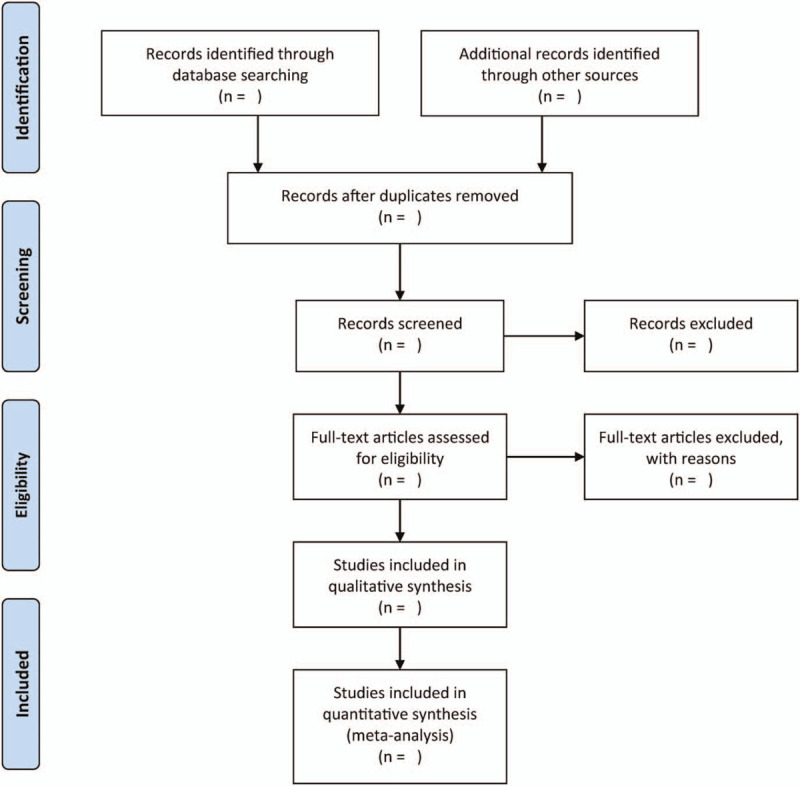
Flowchart of study selection.

We will use 2 independent authors to evaluate titles/abstracts generated using the predetermined search strategy to identify eligibility of titles and abstracts obtained from the above-mentioned electronic databases. They will consider eligibility of these papers according to the inclusion criteria specified above. They will then examine the full-texts to identify relevant studies. We will include all eligible studies in this study and resolve any discrepancies between the authors through consensus. Figure 1 summarizes the whole review process.

#### 3.4.2 Data extraction

We will use 2 independent authors to extract data from the retrieved publications that met the inclusion criteria. We will extract the following data:

1) general information: title, author, country, source, year of publication;
2) intervention characteristics: nursing intervention method (continuous nursing intervention, conventional nursing intervention, or no nursing intervention), duration, intervention time, length of follow-up;
3) participants characteristics: sources (community- or hospital-based), mean age, gender, ethnicity, sampling, number of participants;
4) outcomes: Beck Depression Inventory, Fear of Stroke Recurrence Scale, degree of physical function disability, and Stroke-specific quality of life scale.

#### 3.4.3 Risk of bias assessment

We will use 2 independent authors will examine the risk of bias in all included texts, as per the Cochrane Risk of Bias Tool.^[11]^ We will resolve any discrepancies between the authors through consensus.

#### 3.4.4 Measures of treatment effect

We will use the relative risk and 95% confidence interval to analyze the dichotomous data, and use the mean differences or standardized mean differences and 95% confidence interval to analyze the continuous data.

#### 3.4.5 Dealing with missing data

Where needful, we will contract corresponding authors to verify any missing outcome data.

#### 3.4.6 Assessment of heterogeneity

Furthermore, the Chi^2^ test and the *I*^2^ statistic will be used to assess the statistical heterogeneity across studies. In particular, *P* value less than .1 or *I*^2^ more than 50% will be indicative of statistical heterogeneity. Also, we will utilize the random-effects model to combine data; otherwise, we will use the fixed-effects model to merge data.

#### 3.4.7 Sensitivity analysis

Sensitivity will be undertaken to examine the robustness and stability of our conclusions.

#### 3.4.8 Assessment of reporting biases

Funnel plots will be established to explain the possible small study and publication bias if applicable.

## 4 Discussion

The current study aims to summarize high-quality pieces of evidence to examine the application value of continuous nursing intervention for improving the quality of life among patients suffering from stroke. Accordingly, it will explore a reference for nurses and the improvement of clinical guidelines. We consider that this is the first study to focus on evaluating the application value of continuous nursing intervention for improving the quality of life in patients with stroke. Following an increase in the number of studies that have examined the application value of continuous nursing intervention for improving the quality of life of patients suffering from stroke, it is evident that the results are debatable. To this end, the current study will be carried out to systematically explore the application value of continuous nursing intervention for improving the quality of life among patients suffering from stroke.

## Author contributions

**Conceptualization:** Cai-Yan Han, Xia Yu.

**Data curation:** Cai-Yan Han, Xia Yu.

**Formal analysis:** Cai-Yan Han, Xia Yu.

**Funding acquisition:** Cai-Yan Han, Xia Yu.

**Investigation:** Xia Yu.

**Methodology:** Cai-Yan Han.

**Project administration:** Xia Yu.

**Resources:** Xia Yu.

**Software:** Cai-Yan Han.

**Validation:** Cai-Yan Han.

**Visualization:** Cai-Yan Han, Xia Yu.

**Writing – original draft:** Cai-Yan Han.

**Writing – review & editing:** Cai-Yan Han, Xia Yu.
